# Patient-shared knowledge and information in clinical decision-making: an international survey of the perspectives and experiences of naturopathic practitioners

**DOI:** 10.1186/s12906-023-04087-5

**Published:** 2023-07-18

**Authors:** Amie Steel, Sarah Brand, Matthew Leach, Iva Lloyd, Vicky Ward

**Affiliations:** 1 Australian Research Centre in Complementary and Integrative Medicine, School of Public Health, Faculty of Health, University of Technology Sydney, 235-253 Jones St, Ultimo, 2006 Australia; 2 Nottingham University Hospitals NHS Trust, Nottingham, England; 3 National Centre for Naturopathic Medicine, Southern Cross University, Lismore, Australia; 4 World Naturopathic Federation, Toronto, Canada; 5 Research Unit for Research Utilisation, School of Management, University of St Andrews, Scotland, UK

**Keywords:** Patient-centred care, Shared decision-making, Naturopathy, Knowledge mobilisation, Knowledge translation

## Abstract

**Introduction:**

Most knowledge translation models pay relatively little attention to patient-held knowledge and are largely based on the premise that researchers and clinicians hold all valuable knowledge, and patients are passive recipients of such knowledge. Counter to this clinician- and researcher-centred lens is a growing interest and awareness of patients as experts in their health. While naturopathic medicine is described and experienced as a patient-centred system of traditional medicine, the position of patient-held knowledge is unclear particularly when considered alongside their use of other more objective forms of knowledge such as research evidence.

**Methods:**

This international online cross-sectional survey aimed to explore naturopathic practitioners’ perceptions of the value and contribution of patient-shared knowledge and information within the context of naturopathic clinical consultations.

**Results:**

The survey was completed by 453 naturopathic practitioners (response rate: 74.3%). Approximately two-thirds (68.2%) of respondents reported using information shared by the patient. Most rated ‘information provided by the patient’ as either ‘extremely important’ (60.7%) or ‘very important’ (31.4%) to patients. Highest levels of trust were reported for information provided by the patient (‘completely’: 9.9%; ‘a lot’: 53.6%). Most practitioners indicated they trusted knowledge and information derived from the patient’s personal health history ‘completely’ (*n* = 79; 21.8%) or ‘a lot’ (*n* = 226; 62.4%) from the patient’s perspective of living with a health condition (‘completely’ [*n* = 63, 17.4%]; ‘a lot’ [*n* = 224, 61.9%]). Patients were the highest ranked stakeholder group (mean: 1.5) perceived to influence NP use of patient experience of living with a health condition to inform clinical decision-making.

**Conclusion:**

Researchers and policy makers are increasingly focused on the value of the ‘expert patient’ in clinical decision-making, yet health professionals’ report challenges and, in some cases, resistance to meaningfully engaging with patient-shared knowledge in practice. However, our study has found patient-shared knowledge – inclusive of patient experience of their health condition – is among the knowledge used and trusted by naturopathic practitioners to inform their clinical decision-making. This study both offers insights into the knowledge translation behaviours of an under-researched health profession and provides a novel contribution to the wider aim of adopting patient-shared knowledge into clinical care more generally.

**Supplementary Information:**

The online version contains supplementary material available at 10.1186/s12906-023-04087-5.

## Background

Contemporary health care practice and policy can be challenged by the sometimes conflicting paradigms of evidence-based practice and patient-centred care [1, 2]. While the well-established evidence-based medicine model identifies patient preferences as a key element impacting clinical decision-making [3], the model provides little guidance on how to effectively manage differences between the patient’s preferred treatment approach and use of the best available evidence [2]. In response to this challenge, models of ‘shared decision-making’ have evolved to help guide health professionals through potentially difficult conversations with patients [2]. In parallel, new disciplines and frameworks have emerged to facilitate the translation and implementation of the best available evidence into clinical practice (e.g. implementation science, and knowledge translation) [4]. Although these developments aim to support the research-practice-policy nexus, these models all foreground knowledge from research and clinical practice and pay relatively little attention to patient-held knowledge and are largely based on the premise that researchers and clinicians hold all valuable knowledge, and patients are passive recipients of such knowledge [5]. In fact, there is a worrying trend towards positioning patient behaviour as one of the causes of the gap between evidence and improved clinical outcomes, leading to the development of approaches that encourage patients to unquestioningly follow their health professionals’ advice.

Counter to this clinician- and researcher-centred lens is a growing interest and awareness of patients as experts in their health [6, 7]-a change that is now reflected in statements of both national [8] and international [9] health organisations. The concept of the expert patient recognises that patients possess expert knowledge that is critical to the successful management of their health and illness. Accordingly, knowledge of the patient’s lived experience of an illness, as well as their values, preferences, attitudes, social situation, habits and behaviours, should be integral to the clinical decision-making process [10]. Although many studies have shown that health professionals use a diverse range of knowledge sources to inform their clinical reasoning, patient-shared knowledge is often given relatively low priority in the clinical decision-making process of many health professionals [11–13]. While naturopathic medicine is described and experienced as a patient-centred system of traditional medicine, the position of patient-held knowledge is unclear particularly when considered alongside their use of other more objective forms of knowledge such as research evidence [14]. In response to this gap, this international cross-sectional study aimed to explore naturopathic practitioners’ (NPs) perceptions of the value and contribution of patient-shared knowledge and information within the context of naturopathic clinical consultations.

## Methods

### Design

Cross-sectional survey.

### Aim

This study aimed to investigate NPs’ perceptions of the value and contribution of patient knowledge within the context of naturopathic consultations.

### Setting

Data were collected through an online questionnaire administered throughout the World Naturopath Federation (WNF) global network, inclusive of organisations from 60 countries, between 12th September 2020 and 20th November 2020.

### Participants

The study recruited a self-selected sample of NPs who had been in naturopathic clinical practice within the previous 12 months. Individuals from any country with a naturopathic workforce were invited to participate. Individuals unable to complete the survey in any of the available languages (i.e., English, French, Portuguese, Spanish and German) were excluded. The WNF shared a web-link to the online survey with full member organisations, which then shared the link via direct email with their naturopathic practitioner membership. Both the WNF and the WNF member organisations shared the link through their organisational social media accounts.

### Sample size

The study aimed to recruit a minimum of 385 study participants, which is in line with sample size calculations for descriptive survey research [15]. Participation rate was defined as the number of individuals who completed the survey items pertaining to use of knowledge and information sources to inform clinical decision-making, divided by the number of participants who accessed the information sheet but did not respond to any survey items [16].

### Instrument

The survey design was informed by the knowledge mobilisation framework developed by Ward [17]. The framework identifies domains of questions and categories that can help explore knowledge mobilisation behaviours and attitudes. Some survey items and response options were informed by a report by the National Public Health Institute of Quebec [18], as well as the Evidence-Based practice Attitude and utilization SurvEy (EBASE) validated instrument developed by Leach [19]. Demographic items were informed by previous research conducted through the World Naturopathic Federation and other large naturopathic surveys [20–22].

The questionnaire was administered via Qualtrics™ and included 122 core items, and six adaptive items repeated up to nine times. The degree of repetition of these items was dependent on how a participant responded to the survey item, “Which of the following types of information sources do you employ when providing care to patients?”. The questionnaire items were categorised into seven domains: 1 – demographic and practice characteristics (10 items); 2-practice behaviours (21 items); 3-use of knowledge and information sources (4 items); 4-use of, and attitudes towards, specific knowledge and information sources (6 items repeated adaptively); 5 – experience of patient perceptions and behaviours of using and sharing knowledge and information sources (36 items); 6-perceived stakeholder influence of knowledge use (3 items); and 7-barriers to use of different knowledge types (48 items). This analysis draws on participants’ responses to selected items addressing NPs perceptions and use of patient knowledge and information within clinical decision-making from domains 1, 3, 4, 5 and 6.

#### Domain 1: Demographic and practice characteristics

Items in this domain collected data regarding participants age, gender, country of practice, country of training, time since first qualifying as a naturopathic practitioner, hours per week in clinical practice, patient visits per week, and clinical practice environment.

#### Domain 3: Use of knowledge and information sources

This domain included items that allowed participants to select any of ten information sources and seven knowledge sources they use to inform patient care. They were also provided with the opportunity to report other sources not listed. An item also asked respondents to select any of nine methods used to share their knowledge and to identify any of seven types of knowledge they share with others.

#### Domain 4: Use of, and attitudes towards, specific knowledge and information sources

Respondents were presented with six items exploring their perceptions about the knowledge and information sources they use. These six items were repeated for each information sources selected in items from Domain 3. Specifically, the explored reported and preferred frequency of information source use, knowledge sought from the information source, use and trust of knowledge acquired from information source, and perceived importance to patient that the respondent uses the information source to inform clinical decisions.

#### Domain 5: Experience of patient perceptions and behaviours of using and sharing knowledge and information sources

The fifth Domain investigated participants experiences of the types of knowledge patients share with the respondent (5-point Likert scale: Always – Never), and the perceived importance (5-point Likert scale: Extremely important – Not at all important) and trust (5-point Likert scale: Completely – Not at all) respondents’ place on different knowledge and information sources.

#### Domain 6: Perceived stakeholder influence of knowledge use

Participants were invited to rank a list of ten potential stakeholder groups in order (from 1 as highest to 10 as lowest) of their perceived influence in the participants’ knowledge use. The item was repeated for four knowledge types: knowledge based on research evidence, traditional naturopathic knowledge, knowledge based on patient experience, and knowledge based on clinical experience. The full questionnaire is provided (see Supplementary file 1).

The questionnaire was tested for face validity and technical functionality by three external individuals who were reflective of the target population. Some minor amendments to survey flow and item structure were made based on the feedback of pilot testers.

All participant documents (i.e., invitation email, information sheet, survey) were first drafted in English and then translated into four other languages (i.e., French, Portuguese, Spanish and German) using Qualtrics’ automated translation function. The translations were cross-checked by native language speakers for accuracy and meaning. AS and IL confirmed any changes recommended by translators using Google Translate before edits were applied to the final version. Where applicability of the changes was unclear, a second translator was invited to provide input until consensus was reached. All translations were coordinated by the WNF.

### Recruitment

To maintain participant privacy, no identifying information (e.g., participant name, email address, IP address) was collected by the study instrument and all recruitment was undertaken through third-parties. The WNF shared a web-link to the online survey with full member organisations. The full member organisations subsequently shared the link via direct email with their NP membership. In addition, both the WNF and the WNF member organisations shared the link through social media.

### Data management and analysis

Data were downloaded and stored on the UTS intranet system only accessible to AS. imported to Stata 16.1 (StataCorp LLC) for cleaning, coding and analysis. Missing data were excluded from the analysis. Data on country of location were categorised into World Health Organization defined world regions [23]. Descriptive statistics were performed for all survey items. This included the use of frequencies and percentages for categorical data and means/medians and standard deviations/interquartile ranges for continuous data.

## Results

### Participant characteristics

**Fig. 1:**
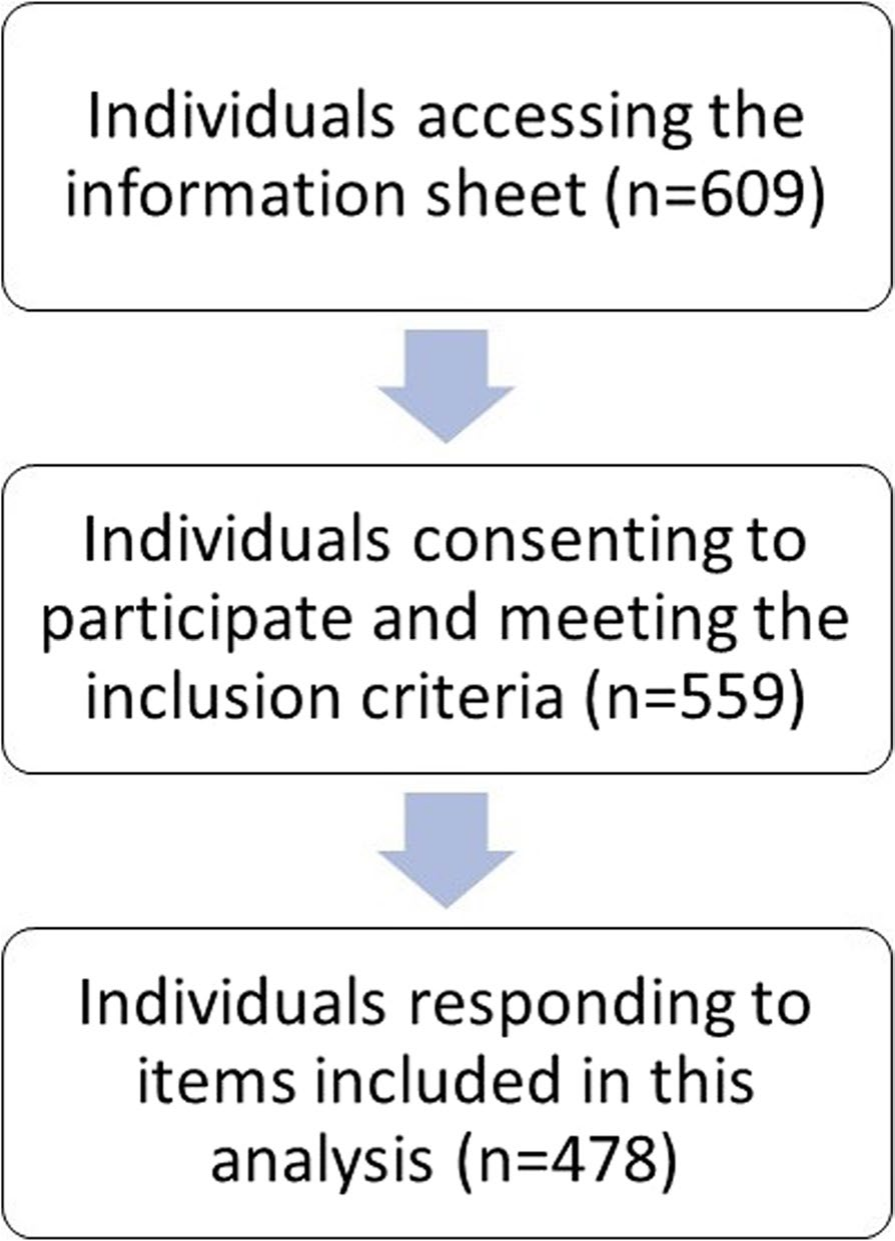
Flow chart demonstrating target population engagement and participant response rate

The survey achieved participation from 74.3% (*n* = 453) of individuals who accessed the information sheet (*n* = 609) (Fig. 1) and median survey completion time was 29.6 min (Q1: 8.1 min; Q3: 63.7 min). Table 1 presents participant demographics and practice characteristics. Participants most commonly identified as female-gendered (73.2%) with a mean age of 45.9 years (SD 12.6). All world regions were represented in the countries where participants were located, with the most common world regions being North America (39.3%), the Western Pacific (22.6%) and Europe (17.5%). The number of years since the participants’ first naturopathic qualification varied with the largest proportion reporting less than five years (24.5%) or between five and ten years (24.9%). Most participants indicated they were in a clinic by themselves (37.2%) although being in a clinic environment with other non-naturopath health professionals – either alongside other NPs (24.6%) or with no other NPs (22.7%) – was also reported by almost half of participants. On average, participants reported working in clinical practice part time (mean: 22.6 h; SD 12.9) and seeing approximately 19 patients per week (mean: 19.3; SD 18.0).

**Table 1:** Characteristics of survey respondents included in analysis (*n* = 453)

	***Mean (SD)***	***Min, max***
Age (*n* = 446)	45.9 (12.6)	23, 81
Gender (*n* = 453)	***n***	**%**
*Male*	124	27.4
*Female*	329	72.6
World Health Region (*n* = 451)		
*North America*	177	39.3
*Latin America*	46	10.2
*Europe*	79	17.5
*Western Pacific*	102	22.6
*Africa/South East Asia/Eastern Mediterranean*	47	10.4
Years since first qualification (*n* = 453)		
*Less than 5 years*	111	24.5
*Between 5 and 10 years*	113	24.9
*Between 11 and 15 years*	73	16.1
*Between 16 and 20 years*	66	14.6
*More than 21 years*	90	19.9
Clinical practice environment (*n* = 452)		
*I am in a clinic by myself*	168	37.2
*I am in a clinic with other health professionals but no other naturopathic practitioners*	103	22.8
*I am in a clinic with other naturopathic practitioners but no other types of health professionals*	34	7.5
*I am in a clinic with other naturopathic practitioners and other health professionals*	114	25.2
*I am in a hospital setting*	10	2.2
*Other settings*	23	5.1
	***Mean (SD)***	***Min, max***
Clinical practice hours per week (*n* = 446)	22.6 (12.9)	1, 60
Patient visits per week (*n* = 450)	19.5 (18.0)	0, 130

### Use, patient-perceived importance and practitioner trust of knowledge and information sources

Participants’ use, perceptions and trust of information sources for clinical decision-making is summarised in Table 2. Information published in scientific journals by researchers (80.4%) or gathered from conferences or other professional events (78.2%) were the two most frequently reported sources of information. Similar rates of use were reported for information published in modern naturopathic clinical textbooks (74.6%), laboratory, pathology or radiology tests (74.0%), or professional journals for clinicians (73.5%). Approximately two-thirds (68.2%) of respondents reported using information shared by the patient.

**Table 2:** Prevalence of use of information sources to inform clinical decision-making by naturopathic practitioners (NP) (*n* = 453), NP perceptions of importance to patient that clinical decisions are informed by the information source, and NP trust of knowledge acquired from the information source

Information source	Use	Perceived importance to patient that clinical decisions are informed by information source^a,b^	Level of practitioner trust of knowledge acquired from information source^a,c^
	*N (%)*	*Mean (SD)*	*Mean (SD)*
Information published in scientific journals by researchers	364 (80.4)	2.45 (0.91)	2.46 (0.58)
Information gathered from conferences or other professional events	354 (78.2)	2.85 (1.03)	2.45 (0.63)
Information published in modern naturopathic clinical textbooks (published in the last 10 years)	338 (74.6)	2.78 (1.01)	2.26 (0.64)
Information from laboratory tests, pathology or radiology tests	335 (74.0)	2.81 (1.88)	1.99 (0.57)
Information published in professional journals for clinicians	333 (73.5)	2.64 (0.91)	2.46 (0.58)
Information provided by the patient	309 (68.2)	1.49 (0.69)	2.31 (0.72)
Information published in general clinical textbooks	296 (65.3)	2.76 (0.98)	2.33 (0.65)
Information from clinical guidelines	248 (54.8)	2.57 (0.99)	2.44 (0.68)
Information provided by product companies	230 (50.8)	3.39 (1.03)	2.88 (0.68)
Information published in traditional naturopathic textbooks (published more than 50 years ago)	193 (42.6)	3.12 (1.03)	2.62 (0.71)

^a^Percentages calculated based on respondents who indicated using the knowledge or information source

^b^Importance scale: 1 = Extremely Important, 5 = Not at all important

^c^Trust scale: 1 = Completely, 5 = Not at all

Participants were asked to rate the perceived importance of different information sources to clinical decision-making, from the patient’s perspective. The mean rating of patient-perceived importance was highest for ‘information provided by the patient’ ( mean: 1.49) followed by information published in scientific journals by researchers (mean: 2.45) and information from clinical guidelines (mean: 2.57) (Table 2). In terms of the extent to which participants trusted different information sources, highest mean levels of trust were reported for knowledge acquired from laboratory, pathology or radiology tests (mean: 1.99) followed by information published in modern naturopathic clinical textbooks (mean: 2.26), information provided by the patient (mean: 2.31) and information published in general clinical textbooks (mean: 2.33) (Table 2).

### Frequency of patient-shared knowledge and practitioner-perceived importance

Participants indicated that patients share knowledge from a range of information sources during the clinical encounter (see Table 3), the highest mean frequency being reported for the patient’s perspective of living with their health condition (mean: 1.65) and their personal health history (mean: 1.85). The participants also attributed the highest mean level of importance to these information sources (perspective of living with their condition: 1.40; personal health history: 1.46). Family health history (mean: 2.22) and conventional medical texts and examinations (mean: 2.30) were also commonly reported by participants as shared by patients with NPs.

**Table 3:** Sources of knowledge or information patients share with naturopathic practitioners

Knowledge or information source	Frequency patients share knowledge or information with naturopathic practitioners (*n* = 371)^a^	Naturopathic practitioner perceived importance of patient-provided knowledge and information sources (*n* = 365)^b^
	*Mean (SD)*	*Mean (SD)*
Perspective of living with their condition	1.65 (0.76)	1.40 (0.63)
Personal health history	1.85 (0.96)	1.46 (0.67)
Family health history	2.22 (1.04)	1.82 (0.82)
Conventional medical tests and examinations	2.30 (0.98)	1.66 (0.70)
General internet sources (e.g., blogs, social media)	2.65 (0.99)	3.95 (0.92)
Other health professionals providing care to the patient	2.95 (0.94)	2.16 (0.77)
Functional examinations or tests (e.g., urine/salivary hormone tests, hair mineral analysis, stool analysis)	3.01 (1.13)	2.13 (0.92)
Informal sources (e.g., family and friends)	3.11 (0.97)	3.64 (1.02)
Broadcast media (e.g., TV, radio)	3.48 (0.99)	4.39 (0.84)
Books	3.53 (0.85)	2.70 (0.91)
Patient advocacy or support groups	4.09 (0.77)	3.26 (0.98)
Published journal articles	4.18 (0.75)	2.32 (0.83)
Government agencies	4.20 (0.68)	3.21 (0.97)
Research organisations	4.25 (0.75)	2.50 (0.87)

^a^Frequency scale: 1 = Always, 5 = Never

^b^Importance scale: 1 = Extremely Important, 5 = Not at all important

According to participants, most patients infrequently (or never) share knowledge or information with NPs from research organisations, government agencies, published journal articles, patient advocacy or support groups, books, broadcast media, and informal sources (Table 3). Of these sources, more than one-half of participants considered published journal articles were rated with the highest mean level of importance (mean: 2.32) while broadcast media, general internet sources, and informal sources were perceived as least important.

### Perceived stakeholder influence on participant use

Stakeholder influence on participant use of knowledge-derived from patient experience.

Table 4 presents the mean ranking of participant perceived stakeholder influence on their using patient experience of living with a health condition to inform clinical decision-making. The highest ranked stakeholder group – indicating frequently ranked as a greater influence-were patients (mean: 1.5), followed by NPs (mean: 3.0) and patient family members (mean: 3.7). Lowest ranked, and thus perceived to be least influential, were third party funders (mean: 9.1), government agencies (mean: 7.9) and other conventional medicine health professionals or organisations (mean: 7.3).

**Table 4:** Order of stakeholder influence on using patient experience of living with their health condition to inform clinical decision-making (1 = highest perceived influence, 10 = lowest perceived influence) (*n* = 337)

Stakeholder group	Mean	SD (Min, Max)
Patients	1.5	1.4 (1,8)
Naturopathic practitioners	3.0	1.3 (1,9)
Patients’ family members	3.7	2.5 (1,10)
Naturopathic professional bodies (e.g., associations)	4.3	1.4 (1,9)
Naturopathic regulatory bodies	5.4	1.7 (1,10)
Researchers	6.3	2.2 (1,10)
Other traditional and complementary medicine health professionals or organisations	6.4	2.3 (1,10)
Other conventional medicine health professionals or organisations	7.3	2.1 (1,10)
Government agencies	7.9	1.6 (2,10)
Third party funders (e.g., health insurers)	9.1	1.2 (1,10)

## Discussion

This study presents the first international examination of NPs’ self-reported use of patient-shared knowledge and information in clinical practice. A key finding from this research is that NPs’ report using a range of knowledge to inform their practice and decision making, of which patient-shared knowledge forms part of this suite of knowledge sources. This finding is supported by globally-used naturopathic clinical texts [24], international position statements by the naturopathic profession [25], and research examining practitioner and patient experiences in Australia, United States and Canada [26–30]; all of which indicate that naturopathy has a legacy of working collaboratively with patients as a core element of practice [26, 27, 31]. This legacy has strong philosophical roots that inform the core principles underpinning naturopathic practice, which are fundamentally patient-centred [32]. Beyond this descriptive exploration of the use of varied knowledge and information sources, an interesting interplay between use and trust in reference to patient knowledge has arisen from this work.

Trust and use are intrinsically linked; trust is established incrementally and based on familiarity developed through previous interactions, or use [33]. NPs’ use of patient-shared knowledge is mid-ranked among the listed knowledge sources, so not all NPs report utilising it; but of those who report using that knowledge, they perceive it as a very trustworthy source. The high level of trust NPs attribute to a patient’s experiential knowledge would indicate that NPs find this knowledge to be reliable and useful in their practice, thereby reinforcing the level of trust this type of knowledge is assigned. The ‘expert patient’ agenda has been prevalent for a number of years in conventional healthcare and the necessity of including patients in decision-making is now enshrined in policy across the world [8, 9]. But healthcare practitioners continue to express reservations about how desirable it is that patients’ experiences are valued, with a view that ‘expert’ has become a synonym for demanding and misinformed patients who are more time consuming to engage with and treat [34]. There are also concerns that involving patients can at times lead to reduced patient satisfaction with consultations and treatment [35, 36]. There appear to be no such concerns in naturopathic practice based on the evidence presented here and that, in this context, patient experiential knowledge and practitioner knowledge can successfully work together to the mutual benefit of both parties.

The trust NPs place on patient-shared experiential knowledge may, in fact, indicate a crucial difference between NPs and conventional healthcare professions. Specifically, our study suggests that NPs may be more comfortable and willing to use patient-shared experiential knowledge to inform their decision-making compared with conventional healthcare professionals; although, we acknowledge that this is somewhat speculative and does warrant further exploration. Work on epistemic injustice does however highlight how patients in conventional healthcare contexts are often viewed as unreliable and their interpretations of events suspect [37, 38]. This gives epistemically privileged health professionals dispensation to disregard elements of patient knowledge that they find uncomfortable or difficult to use. In conventional healthcare practice, professionals often dismiss patient-shared experiential knowledge in favour of biomedically-based clinical knowledge [39], particularly when there are conflicts between the two. However, our study found NPs perceived patient knowledge to be more trustworthy than other sources of knowledge such as clinical guidelines or professional journal publications, which are usually highly valued in conventional healthcare practice given the EBP movement. This high level of trust NPs attribute to patient knowledge may be a potential foundation for an easier exchange of expertise with regards to decision-making between NPs and patients, which again is at odds with conventional healthcare practice.

Of the knowledge that patients share, NPs perceived the patient’s perspective of living with their condition and personal health history – their experiential knowledge – to be particularly important. Despite being shared frequently by patients, second-hand acquired knowledge from family, friends, broadcast media or general internet sources were seen by our participants to be much less important, even though it may be quasi-biomedical in nature. However, socially constructed perspective of knowledge [40] would dispute naturopathic practitioners’ ability to distinguish types of knowledge in this way. Rather, this perspective asserts knowledge is contingent on the beliefs and perceptions of social actors, being fundamentally associated with the individual knower and their context. Therefore, any experiential knowledge shared by patients will be, to some extent, influenced by friends, family, and any other knowledge that they have encountered. As such, NPs distinction between the types of knowledge that patients share, and their relative importance, may be a false one. The degree to which NPs are aware of such potential conflicts when drawing on patient-shared knowledge to inform their clinical decisions requires further research. Such research may benefit from considering use of patient-shared knowledge through the framework of different types of knowledge use including instrumental (‘knowledge-driven’, ‘problem-solving’), conceptual (‘interactive’, ‘enlightenment’, ‘intellectual) and strategic (‘political’, tactical’) uses [41]. In fact, a closer study of the dynamics of knowledge sharing and use within naturopathic consultations may benefit not only the users and practitioners of naturopathy but may offer insights to improve and strengthen patient-centred communication and health service delivery more broadly.

### Limitations

As this study employed a convenience sampling method the results are at risk of sampling bias. However, the absence of definitive lists of naturopathic practitioners in many of the countries through which recruitment was conducted precluded other sampling methods. The study is also subject to recall bias due to the retrospective nature of the questions and the potential for participants to provide responses seen as desirable. Another limitation of this research, particularly in relation to a discussion of patient-shared knowledge, is that only the perspectives of NPs was obtained. Patients were not consulted in this study. This reveals an underlying assumption that NPs are the gatekeepers of knowledge used in clinical decision-making. Despite the previous discussion indicating that conventional healthcare professionals may have something to learn from naturopathic practice in relation to the use of patient experiential knowledge, an assumption that practitioners determine which knowledge is used in decision-making does nothing to challenge extant power relations within wider healthcare practice. While previous research investigating patient perceptions and experiences of naturopathic care does support the findings of our study, it was not focused specifically on patient-shared knowledge sharing and use, but rather experiences of patient-centred care more generally [26, 27]. It is also important to acknowledge that the survey questions did not specify specific health conditions and it is possible that participants use knowledge and information differently for different health conditions, particularly in the case of naturopathy which has demonstrated a complexity-approach to clinical reasoning [21, 42]. Similarly, the survey data does not ascertain the degree to which the participants were critically appraising the knowledge and information they gathered or whether they were using the best available evidence, in line with evidence-based practice principles.

### Future research directions

This is an underexamined topic and as such there are numerous areas identified through this exploratory research that would benefit from further researcher attention. One such area is the methods and techniques used by NPs when considering patient-shared knowledge and information into clinical decision-making. Due to the current evidence gaps with regards to this line of enquiry, such future research would be best undertaken using an exploratory mixed-methods approach that employs qualitative research methods (e.g., interviews and focus groups) to provide an in-depth examination of practitioner perspectives followed by quantitative methods (e.g., survey research or network mapping) to statistically measure identified trends and patterns. Further research could also employ a variety of research methods to explore this topic from the perspective the patient and their views on knowledge and its use within naturopathic practice. There is also potential value in exploring whether NPs engage with patient-provided knowledge differently when providing care to different illness populations. Beyond naturopathy, there is a need for future research to explore this topic within the context of other health professions.

## Conclusions

Researchers and policy makers are increasingly focused on the value of the ‘expert patient’ in clinical decision-making, yet health professionals’ report challenges and, in some cases, resistance to meaningfully engaging with patient-shared knowledge in practice. However, our study has found patient-shared knowledge – inclusive of patient experience of their health condition – is among the knowledge used and trusted by NPs to inform their clinical decision-making. As the scholarship surrounding society’s use of knowledge continues to grow, so does the need to better understand how the interplay between different knowledge sources, including patient-shared information, manifests in the clinical decision-making process of all health professions, including naturopathy. As such, this study both offers insights into the knowledge translation behaviours of an under-researched health profession and provides a novel contribution to the wider aim of adopting patient-shared knowledge into clinical care more generally.

## Supplementary Information

**Additional file 1.**

## Data Availability

The data that support the findings of this study are available from the corresponding author upon reasonable request.
