# Supplementary material for: Patient-shared knowledge and information in clinical decision-making: an international survey of the perspectives and experiences of naturopathic practitioners
Source: BMC Complement Med Ther. 2023 Jul 18;23:247. doi: 10.1186/s12906-023-04087-5 (PMC10354883; doi:10.1186/s12906-023-04087-5)
Supplement: Supplementary file 1 — Additional file 1. [file 12906_2023_4087_MOESM1_ESM.docx]

Naturopathic practitioners' approach to knowledge mobilisation and translation

Start of Block: Information sheet

QID201 **INFORMATION SHEET AND CONSENT FORM FOR ONLINE SURVEYS**   **ETH20-5273 – Naturopathic practitioners’ approach to the mobilisation and translation of knowledge in clinical practice**    **What is the research study about?** The purpose of this research/online survey is to describe the approach naturopathic practitioners take to using and sharing different types of knowledge and information in clinical practice. You have been invited to participate because you identify as a naturopathic practitioner in clinical practice.      **Who is conducting this research?** My name is Dr Amie Steel and I am a naturopath and researcher at University of Technology Sydney (UTS), Australia.  I am collaborating with Dr Matthew Leach (Southern Cross University, Australia), Dr Caragh Brosnan (University of Newcastle, Australia) and Dr Vicky Ward (University of St Andrew’s, Scotland).   **Inclusion/Exclusion Criteria** Before you decide to participate in this research study, we need to ensure that it is ok for you to take part. Individuals who have a recognised naturopathic qualification in the country where they are located and currently or recently in clinical practice as a naturopathic practitioner are invited to participate. This includes individuals on temporary leave from practice due to government restrictions in response to COVID or parental leave. Individuals who have discontinued clinical practice and have not provided clinical care for the previous 12 months or longer are excluded.   **Do I have to take part in this research study?** Participation in this study is voluntary. It is completely up to you whether or not you decide to take part. If you decide to participate, I will invite you to read the information carefully (ask questions if necessary) and complete an online questionnaire.   You can change your mind at any time and stop completing the survey without consequences.   **Are there any risks/inconvenience?** This survey will take between 20 and 40 minutes to complete. We do not expect this questionnaire to cause any harm or discomfort.   In acknowledgement of the time you would commit to the participate in this study, you will be given the opportunity to enter a prize draw to win one of five naturopathic clinical textbooks. To enter the prize draw you will be asked to follow a weblink to a new survey where you can enter your name and email address. The prize draw survey is completely separate from the main survey and none of your personal details will be linked to your survey responses. The prize winners will be notified within one month after the survey is closed.   **What will happen to information about me?** The online questionnaire is accessed by responding to the items at the bottom of this page. By clicking ‘Yes’ you consent to the research team collecting and using personal information about you for the research project. All this information will be treated confidentially and collected anonymously. We plan to publish the results of this study in a peer-reviewed journal publication and provide a summary document for use by national and international naturopathic organisations to share with stakeholders in their jurisdiction. **What if I have concerns or a complaint?** If you have concerns about the research that you think I can help you with, please feel free to contact me on amie.steel@uts.edu.au.  If you would like to verify details about the research team and the project you may contact Dr Iva Lloyd, President of the World Naturopathic Federation on president@worldnaturopathicfederation.org, or the professional association in your country. If you would like to talk to someone who is not connected with the research, you may contact the Research Ethics Officer on +61 2 9514 9772 or Research.ethics@uts.edu.au and quote this number ETH20-5273

QID206 I have read the information concerning this research and understand what is required of me

- Yes (1)
- No (2)

Display This Question:

If I have read the information concerning this research and understand what is required of me = No

QID207 If you have concerns about the research that you think I can help you with, please feel free to contact me on amie.steel@uts.edu.au.  If you would like to verify details about the research team and the project you may contact Dr Iva Lloyd, President of the World Naturopathic Federation on president@worldnaturopathicfederation.org, or the professional association in your country.If you would like to talk to someone who is not connected with the research, you may contact the Research Ethics Officer on +61 2 9514 9772 or Research.ethics@uts.edu.au and quote this number ETH20-5273

Display This Question:

If I have read the information concerning this research and understand what is required of me = Yes

QID205 Do you consent to participate in this study?

- Yes (1)
- No (2)

Skip To: End of Survey If Do you consent to participate in this study? = No

End of Block: Information sheet

Start of Block: Screening questions

| 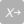 |
| --- |

QID6 Are you currently in clinical practice?

- Yes (1)
- No (0)

Display This Question:

If Are you currently in clinical practice? = No

| 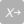 |
| --- |

QID194 What best describes your current circumstances regarding clinical practice?

- I have taken temporary leave from clinical practice within the last 12 months (1)
- I have retired from clinical practice within the last 12 months (2)
- I have not been in clinical practice for more than 12 months (3)

End of Block: Screening questions

Start of Block: Get comfortable

Q155 Thank you for agreeing to participate in this important survey. You will need 20- 40 minutes to complete the survey questions. 


If you need to stop for a while you can save your responses and continue later, but you will need to use the same browser (e.g. Google Chrome, Firefox, Edge) on the same computer or device. 


Otherwise please: make yourself a herbal tea, get comfortable, and continue when you are ready.

End of Block: Get comfortable

Start of Block: Demographics and practice behaviours

| 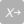 |
| --- |

QID1 What is your gender?

- Male (1)
- Female (2)
- Non-binary (3)

| 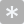 |
| --- |

QID5 What is your current age in years?

________________________________________________________________

| 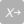 |
| --- |

QID3 What country are your primarily located in?

▼ Australia (35) ... Other (68)

Display This Question:

If What country are your primarily located in? = Other

QID54 Please write the country where you are primarily located?

________________________________________________________________

| 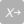 |
| --- |

QID4 In what country did you receive your first naturopathic qualification?

▼ Australia (69) ... Other (102)

Display This Question:

If In what country did you receive your first naturopathic qualification? = Other

QID55 Please write the country where you received your first naturopathic qualification

________________________________________________________________

| 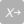 |
| --- |

QID62 How long ago did you first qualify as a naturopathic practitioner?

- Less than 5 years (1)
- Between 5 and 10 years (2)
- Between 11 and 15 years (3)
- Between 16 and 20 years (4)
- More than 21 years (5)

| 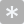 |
| --- |

QID195 On average, how many hours are you in clinical practice per week?

________________________________________________________________

| 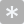 |
| --- |

QID7 On average, how many patient visits do you have in clinical practice each week?

________________________________________________________________

| 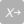 |
| --- |

QID8 Which of the following best describes your clinical practice environment?

- I am in a clinic by myself (1)
- I am in a clinic with other health professionals but no other naturopathic practitioners (2)
- I am in a clinic with other naturopaths but no other types of health professionals (3)
- I am in in a clinic with other naturopathic practitioners and other health professionals (4)
- I am in a hospital setting (5)
- Other (please give details) (6) ________________________________________________

| Page Break |  |
| --- | --- |

QID96 The following questions relate to treatment practices you may employ, either directly or indirectly, as part of the care given to your patients.

| 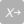 |
| --- |

QID74 How frequently do you prescribe, suggest or recommend **dietary changes** to your patients?

- Always (1)
- Most of the time (2)
- About half the time (3)
- Sometimes (4)
- Never (5)

| 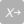 |
| --- |

QID75 How frequently do you prescribe, suggest or recommend **lifestyle behaviour changes (e.g. exercise, yoga and meditation, smoking, alcohol intake)** to your patients?

- Always (1)
- Most of the time (2)
- About half the time (3)
- Sometimes (4)
- Never (5)

| 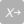 |
| --- |

QID76 How frequently do you prescribe, suggest or recommend **herbal medicines** to your patients?

- Always (1)
- Most of the time (2)
- About half the time (3)
- Sometimes (4)
- Never (5)

| 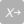 |
| --- |

QID77 How frequently do you prescribe, suggest or recommend **nutritional supplements** to your patients?

- Always (1)
- Most of the time (2)
- About half the time (3)
- Sometimes (4)
- Never (5)

| 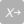 |
| --- |

QID78 How frequently do you perform, prescribe, suggest or recommend **acupuncture** to your patients?

- Always (1)
- Most of the time (2)
- About half the time (3)
- Sometimes (4)
- Never (5)

| 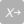 |
| --- |

QID79 How frequently do you perform, prescribe, suggest or recommend **manual therapies** to your patients?

- Always (1)
- Most of the time (2)
- About half the time (3)
- Sometimes (4)
- Never (5)

| 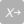 |
| --- |

QID80 How frequently do you perform, prescribe, suggest or recommend **homeopathy** to your patients?

- Always (1)
- Most of the time (2)
- About half the time (3)
- Sometimes (4)
- Never (5)

| 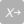 |
| --- |

QID81 How frequently do you perform, prescribe, suggest or recommend **counselling and psychotherapy** to your patients?

- Always (1)
- Most of the time (2)
- About half the time (3)
- Sometimes (4)
- Never (5)

| 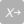 |
| --- |

QID82 How frequently do you prescribe, suggest or recommend **other energetic medicines (e.g. flower essences)** to your patients?

- Always (1)
- Most of the time (2)
- About half the time (3)
- Sometimes (4)
- Never (5)

| 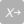 |
| --- |

QID83 How frequently do you perform, prescribe, suggest or recommend **hydrotherapy** to your patients?

- Always (1)
- Most of the time (2)
- About half the time (3)
- Sometimes (4)
- Never (5)

| 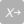 |
| --- |

QID84 How frequently do you perform, prescribe, suggest or recommend **other traditional medicine systems (e.g. Ayurveda, Traditional Chinese medicine)** to your patients?

- Always (1)
- Most of the time (2)
- About half the time (3)
- Sometimes (4)
- Never (5)

| 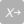 |
| --- |

QID85 How frequently do you perform, prescribe, suggest or recommend **injection therapies (e.g. intravenous injections, intramuscular injections)** to your patients?

- Always (1)
- Most of the time (2)
- About half the time (3)
- Sometimes (4)
- Never (5)

| Page Break |  |
| --- | --- |

QID97 The following questions relate to topics you may discuss with your patients during consultations

| 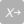 |
| --- |

QID87 How frequently do you discuss **diet and nutrition** with your patients?

- Always (1)
- Most of the time (2)
- About half the time (3)
- Sometimes (4)
- Never (5)

| 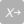 |
| --- |

QID88 How frequently do you discuss **sleep** with your patients?

- Always (1)
- Most of the time (2)
- About half the time (3)
- Sometimes (4)
- Never (5)

| 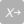 |
| --- |

QID90 How frequently do you discuss **stress management**with your patients?

- Always (1)
- Most of the time (2)
- About half the time (3)
- Sometimes (4)
- Never (5)

| 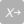 |
| --- |

QID91 How frequently do you discuss **physical activity and fitness**with your patients?

- Always (1)
- Most of the time (2)
- About half the time (3)
- Sometimes (4)
- Never (5)

| 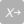 |
| --- |

QID92 How frequently do you discuss **pharmaceuticals and other medication**with your patients?

- Always (1)
- Most of the time (2)
- About half the time (3)
- Sometimes (4)
- Never (5)

| 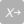 |
| --- |

QID93 How frequently do you discuss **substance use (e.g. tobacco, alcohol, illicit drugs)**with your patients?

- Always (1)
- Most of the time (2)
- About half the time (3)
- Sometimes (4)
- Never (5)

| 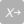 |
| --- |

QID94 How frequently do you discuss **counselling and mental health**with your patients?

- Always (1)
- Most of the time (2)
- About half the time (3)
- Sometimes (4)
- Never (5)

| 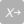 |
| --- |

QID95 How frequently do you discuss **environmental health and toxins**with your patients?

- Always (1)
- Most of the time (2)
- About half the time (3)
- Sometimes (4)
- Never (5)

| 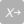 |
| --- |

QID197 How frequently do you discuss **relationships and support from others**with your patients?

- Always (1)
- Most of the time (2)
- About half the time (3)
- Sometimes (4)
- Never (5)

End of Block: Demographics and practice behaviours

Start of Block: Use of knowledge and information sources

| 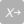 |
| --- |

QID13 Which of the following **information sources** do you use to **inform the care** you provide to your patients? (select all that apply)

- information published in scientific journals by researchers (1)
- information published in professional journals for clinicians (2)
- information published in general clinical text books (3)
- information published in modern naturopathic clinical text books (published in the last 10 years) (4)
- information published in traditional naturopathic clinical text books (published more than 50 years ago) (5)
- information provided by product companies (6)
- information gathered from conferences or other professional events (7)
- information from clinical guidelines (8)
- information from laboratory tests, pathology or radiology tests (9)
- information provided by the patient (10)
- Other (please describe) (11) ________________________________________________

| 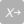 |
| --- |

QID50 Which of the following types of **knowledge** do you use to **inform the care** you provide to your patients? (select all that apply)

- knowledge developed during your initial clinical training (1)
- knowledge developed through continuing professional education delivered by an expert clinician (2)
- knowledge developed through continuing professional education delivered by a researcher (3)
- knowledge developed through discussions with professional peers (4)
- knowledge developed through discussions with a mentor or expert (5)
- knowledge developed through clinical experience (6)
- knowledge developed through consideration of the patient's unique needs (7)
- other (please describe) (8) ________________________________________________

| 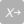 |
| --- |

QID14 What methods do you use to share your own knowledge? (select all that apply)

- producing information to be published in scientific journal articles (1)
- producing information to be published in naturopathic journal articles (2)
- producing information to be published in general clinical text books (3)
- producing information to be published in modern naturopathic clinical text books (4)
- producing information for patients (e.g. information handouts, newsletters) (5)
- producing information for product companies (6)
- producing information delivered through continuing professional education events for other clinicians (7)
- producing information delivered through clinical training for naturopathic students (8)
- producing information for the general public (e.g social media, blogs, community talks, magazine articles) (9)

| 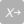 |
| --- |

QID63 Which of the following types of **knowledge** do you **share with others** in your role as a naturopath? (select all that apply)

- knowledge developed during your initial clinical training (1)
- knowledge developed through continuing professional education delivered by an expert clinician (2)
- knowledge developed through continuing professional education delivered by a researcher (4)
- knowledge developed through discussions with professional peers (6)
- knowledge developed through discussions with a mentor or expert (7)
- knowledge developed through clinical experience (8)
- knowledge developed through consideration of the patient's unique needs (7)
- other (please describe) (8) ________________________________________________

End of Block: Use of knowledge and information sources

Start of Block: Use of, and attitudes towards, specific knowledge and information sources

QID66 These questions specifically relate to your perceptions and experiences using**${lm://Field/1}** in your clinical role.

| 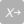 |
| --- |

QID16 How often **do** you use ${lm://Field/1} as part of your clinical practice?

- Always (1)
- Most of the time (2)
- About half the time (3)
- Sometimes (4)
- Never (5)

| 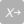 |
| --- |

QID17 How often **would you prefer** to use ${lm://Field/1} as part of your clinical practice?

- Always (1)
- Most of the time (2)
- About half the time (3)
- Sometimes (4)
- Never (5)

| 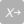 |
| --- |

QID18 What sort of knowledge do you seek from ${lm://Field/1}?

- How a treatment works (1)
- How a treatment might benefit my patient (2)
- The safety of a treatment for my patient (3)
- Understanding of my patient's health condition (4)
- Understanding about treatments my patient has been prescribed by another health professional (5)
- Other (please describe) (6) ________________________________________________

| 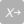 |
| --- |

QID19 How do you use the knowledge you acquire from ${lm://Field/1}?

- To share with my patient (1)
- To share with other health professionals (2)
- To inform my own clinical decisions for an individual patient (3)
- To share with the general community (4)
- To develop clinical practice guidelines or policies for a group of patients (5)
- To produce useful research or scientific knowledge (6)
- Other (please describe) (7) ________________________________________________

| 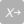 |
| --- |

QID20 How much do you trust knowledge you acquire from ${lm://Field/1}?

- Completely (1)
- A lot (2)
- A moderate amount (3)
- A little (4)
- None at all (5)

| 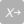 |
| --- |

QID22 In your opinion, how important is it to your patient that you use ${lm://Field/1} to inform your clinical decision-making?

- Extremely important (1)
- Very important (2)
- Moderately important (3)
- Slightly important (4)
- Not at all important (5)

End of Block: Use of, and attitudes towards, specific knowledge and information sources

Start of Block: Encouragement 1

QID190 You are half way there!! Please keep going - your answers are so important to naturopathic practitioners everywhere!


And remember - you can take a break if you need to and come back later. You just need to return to the survey link using the same internet browser (e.g. Google Chrome, Firefox, Edge) on the same computer or device.

End of Block: Encouragement 1

Start of Block: Preceptions about knowledge and information sources

QID69 The following questions relate to **knowledge or information your patient may share with you** in the context of you providing them clinical care. This may include knowledge or information they volunteer, or that you request of them.

| 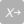 |
| --- |

QID98 In your experience, how often do patients share with you knowledge or information drawn from **their perspective of living with their health condition?**

- Always (1)
- Most of the time (2)
- About half the time (3)
- Sometimes (4)
- Never (5)

| 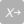 |
| --- |

QID198 In your experience, how often do patients share with you knowledge or information drawn from **books?**

- Always (1)
- Most of the time (2)
- About half the time (3)
- Sometimes (4)
- Never (5)

| 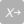 |
| --- |

QID99 In your experience, how often do patients share with you knowledge or information drawn from **general internet sources (e.g. blogs, social media)?**

- Always (1)
- Most of the time (2)
- About half the time (3)
- Sometimes (4)
- Never (5)

| 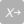 |
| --- |

QID100 In your experience, how often do patients share with you knowledge or information drawn from **published journal articles?**

- Always (1)
- Most of the time (2)
- About half the time (3)
- Sometimes (4)
- Never (5)

| 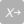 |
| --- |

QID101 In your experience, how often do patients share with you knowledge or information drawn from **research organisations?**

- Always (1)
- Most of the time (2)
- About half the time (3)
- Sometimes (4)
- Never (5)

| 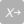 |
| --- |

QID103 In your experience, how often do patients share with you knowledge or information drawn from **government agencies?**

- Always (1)
- Most of the time (2)
- About half the time (3)
- Sometimes (4)
- Never (5)

| 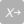 |
| --- |

QID104 In your experience, how often do patients share with you knowledge or information drawn from **other health professionals involved in their care?**

- Always (1)
- Most of the time (2)
- About half the time (3)
- Sometimes (4)
- Never (5)

| 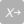 |
| --- |

QID105 In your experience, how often do patients share with you knowledge or information drawn from **patient advocacy or support groups?**

- Always (1)
- Most of the time (2)
- About half the time (3)
- Sometimes (4)
- Never (5)

| 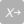 |
| --- |

QID106 In your experience, how often do patients share with you knowledge or information drawn from **informal sources (e.g. family and friends)?**

- Always (1)
- Most of the time (2)
- About half the time (3)
- Sometimes (4)
- Never (5)

| 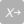 |
| --- |

QID107 In your experience, how often do patients share with you knowledge or information drawn from **broadcast media (e.g. TV, radio)?**

- Always (1)
- Most of the time (2)
- About half the time (3)
- Sometimes (4)
- Never (5)

| 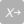 |
| --- |

QID108 In your experience, how often do patients share with you knowledge or information drawn from **their personal health history?**

- Always (1)
- Most of the time (2)
- About half the time (3)
- Sometimes (4)
- Never (5)

| 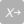 |
| --- |

QID109 In your experience, how often do patients share with you knowledge or information drawn from **their family health history?**

- Always (1)
- Most of the time (2)
- About half the time (3)
- Sometimes (4)
- Never (5)

| 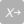 |
| --- |

QID110 In your experience, how often do patients share with you knowledge or information drawn from **conventional medical examinations or tests?**

- Always (1)
- Most of the time (2)
- About half the time (3)
- Sometimes (4)
- Never (5)

| 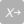 |
| --- |

QID203 In your experience, how often do patients share with you knowledge or information drawn from **functional examinations or**tests (e.g. urine/salivary hormone tests, hair mineral analysis, stool analysis)?

- Always (1)
- Most of the time (2)
- About half the time (3)
- Sometimes (4)
- Never (5)

| Page Break |  |
| --- | --- |

QID123 The following questions relate to the importance you place on some sources of knowledge and information when making decisions about the care you provide to a patient

| 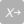 |
| --- |

QID111  When you are making decisions about the care you provide to a patient, how important to you is knowledge or information from **the patient's perspective of living with their health condition?**

- Extremely important (1)
- Very important (2)
- Moderately important (3)
- Slightly important (4)
- Not at all important (5)

| 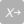 |
| --- |

QID199  When you are making decisions about the care you provide to a patient, how important to you is knowledge or information from **books?**

- Extremely important (1)
- Very important (2)
- Moderately important (3)
- Slightly important (4)
- Not at all important (5)

| 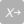 |
| --- |

QID112  When you are making decisions about the care you provide to a patient, how important to you is knowledge or information from **general internet sources (e.g. blogs, social media)?**

- Extremely important (1)
- Very important (2)
- Moderately important (3)
- Slightly important (4)
- Not at all important (5)

| 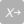 |
| --- |

QID113  When you are making decisions about the care you provide to a patient, how important to you is knowledge or information from **published journal articles?**

- Extremely important (1)
- Very important (2)
- Moderately important (3)
- Slightly important (4)
- Not at all important (5)

| 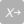 |
| --- |

QID114  When you are making decisions about the care you provide to a patient, how important to you is knowledge or information from **research organisations?**

- Extremely important (1)
- Very important (2)
- Moderately important (3)
- Slightly important (4)
- Not at all important (5)

| 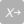 |
| --- |

QID116  When you are making decisions about the care you provide to a patient, how important to you is knowledge or information from **government agencies?**

- Extremely important (1)
- Very important (2)
- Moderately important (3)
- Slightly important (4)
- Not at all important (5)

| 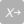 |
| --- |

QID117  When you are making decisions about the care you provide to a patient, how important to you is knowledge or information from **other health professionals involved in their care?**

- Extremely important (1)
- Very important (2)
- Moderately important (3)
- Slightly important (4)
- Not at all important (5)

| 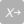 |
| --- |

QID124  When you are making decisions about the care you provide to a patient, how important to you is knowledge or information from **patient advocacy or support groups?**

- Extremely important (1)
- Very important (2)
- Moderately important (3)
- Slightly important (4)
- Not at all important (5)

| 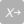 |
| --- |

QID118  When you are making decisions about the care you provide to a patient, how important to you is knowledge or information from **informal sources (e.g family and friends)?**

- Extremely important (1)
- Very important (2)
- Moderately important (3)
- Slightly important (4)
- Not at all important (5)

| 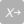 |
| --- |

QID119  When you are making decisions about the care you provide to a patient, how important to you is knowledge or information from **broadcast media (e.g. TV, radio)?**

- Extremely important (1)
- Very important (2)
- Moderately important (3)
- Slightly important (4)
- Not at all important (5)

| 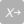 |
| --- |

QID120  When you are making decisions about the care you provide to a patient, how important to you is knowledge or information from **their personal health history?**

- Extremely important (1)
- Very important (2)
- Moderately important (3)
- Slightly important (4)
- Not at all important (5)

| 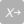 |
| --- |

QID121  When you are making decisions about the care you provide to a patient, how important to you is knowledge or information from **their family health history?**

- Extremely important (1)
- Very important (2)
- Moderately important (3)
- Slightly important (4)
- Not at all important (5)

| 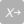 |
| --- |

QID122  When you are making decisions about the care you provide to a patient, how important to you is knowledge or information from **medical examinations or tests?**

- Extremely important (1)
- Very important (2)
- Moderately important (3)
- Slightly important (4)
- Not at all important (5)

| 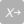 |
| --- |

QID204  When you are making decisions about the care you provide to a patient, how important to you is knowledge or information from functional examinations or tests (e.g. urine/salivary hormone tests, hair mineral analysis, stool analysis)**?**

- Extremely important (1)
- Very important (2)
- Moderately important (3)
- Slightly important (4)
- Not at all important (5)

| Page Break |  |
| --- | --- |

QID125 The following questions relate to how much you trust some sources of knowledge and information when you are making decisions about the care you provide to a patient

| 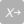 |
| --- |

QID126 How much do you trust knowledge or information from **the patient's perspective of living with their health condition?**

- Completely (1)
- A lot (2)
- A moderate amount (3)
- A little (4)
- Not at all (5)

| 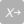 |
| --- |

QID200 How much do you trust knowledge or information from **books?**

- Completely (1)
- A lot (2)
- A moderate amount (3)
- A little (4)
- Not at all (5)

| 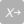 |
| --- |

QID127 How much do you trust knowledge or information from **general internet sources (e.g. blogs, social media)?**

- Completely (1)
- A lot (2)
- A moderate amount (3)
- A little (4)
- Not at all (5)

| 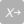 |
| --- |

QID128 How much do you trust knowledge or information from **published journal articles?**

- Completely (1)
- A lot (2)
- A moderate amount (3)
- A little (4)
- Not at all (5)

| 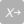 |
| --- |

QID129 How much do you trust knowledge or information from **research organisations?**

- Completely (1)
- A lot (2)
- A moderate amount (3)
- A little (4)
- Not at all (5)

| 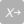 |
| --- |

QID130 How much do you trust knowledge or information from **government agencies?**

- Completely (1)
- A lot (2)
- A moderate amount (3)
- A little (4)
- Not at all (5)

| 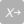 |
| --- |

QID131 How much do you trust knowledge or information from **other health professionals involved in their care?**

- Completely (1)
- A lot (2)
- A moderate amount (3)
- A little (4)
- Not at all (5)

| 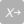 |
| --- |

QID132 How much do you trust knowledge or information from **patient advocacy or support groups?**

- Completely (1)
- A lot (2)
- A moderate amount (3)
- A little (4)
- Not at all (5)

| 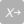 |
| --- |

QID133 How much do you trust knowledge or information from **informal sources (e.g family and friends)?**

- Completely (1)
- A lot (2)
- A moderate amount (3)
- A little (4)
- Not at all (5)

| 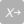 |
| --- |

QID134 How much do you trust knowledge or information from **broadcast media (e.g. TV, radio)?**

- Completely (1)
- A lot (2)
- A moderate amount (3)
- A little (4)
- Not at all (5)

| 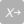 |
| --- |

QID135 How much do you trust knowledge or information from from **their personal health history?**

- Completely (1)
- A lot (2)
- A moderate amount (3)
- A little (4)
- Not at all (5)

| 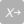 |
| --- |

QID136 How much do you trust knowledge or information from **their family health history?**

- Completely (1)
- A lot (2)
- A moderate amount (3)
- A little (4)
- Not at all (5)

| 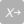 |
| --- |

QID137 How much do you trust knowledge or information from **conventional medical examinations or tests?**

- Completely (1)
- A lot (2)
- A moderate amount (3)
- A little (4)
- Not at all (5)

| 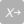 |
| --- |

QID202 How much do you trust knowledge or information from **functional medical examinations or tests (e.g. urine/salivary hormone tests, hair mineral analysis, stool analysis)?**

- Completely (1)
- A lot (2)
- A moderate amount (3)
- A little (4)
- Not at all (5)

| Page Break |  |
| --- | --- |

End of Block: Preceptions about knowledge and information sources

Start of Block: Encouragement 2

QID191 Keep going - the results from this survey will be shared throughout the naturopathic profession. Every completed question matters!


If you can't keep progressing right now, feel free to come back and finish the survey later. You can do this by clicking on the survey link using the same internet browser (e.g. Google Chrome, Firefox, Edge) on the same computer or device.

End of Block: Encouragement 2

Start of Block: Perceived stakeholder influence of knowledge use

QID64 The following items ask you to rank a list of stakeholder groups in order of importance. 


Please 'click and drag' each option until they are placed in the order you consider appropriate, placing the most important group at the top of the list and the least important at the bottom of the list.

| 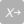 |
| --- |

QID58 In your opinion, in what order do the following groups influence your decision to use **knowledge based on research evidence**in your clinical practice?

______ Patients (1)

______ Patients' family members (2)

______ Naturopathic practitioners (3)

______ Naturopathic professional bodies (e.g. associations) (4)

______ Naturopathic regulatory bodies (5)

______ Government agencies (6)

______ Researchers (7)

______ Third party funders (e.g. health insurers) (8)

______ Other conventional medicine health professionals or organisations (9)

______ Other traditional and complementary medicine health professionals or organisations (10)

| 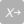 |
| --- |

QID59 In your opinion, in what order do the following groups influence your decision to use **traditional naturopathic knowledge** in your clinical practice?

______ Patients (1)

______ Patients' family members (2)

______ Naturopathic practitioners (3)

______ Naturopathic professional bodies (e.g. associations) (4)

______ Naturopathic regulatory bodies (5)

______ Government agencies (6)

______ Researchers (7)

______ Third party funders (e.g. health insurers) (8)

______ Other conventional medicine health professionals or organisations (9)

______ Other traditional and complementary medicine health professionals or organisations (10)

| 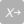 |
| --- |

QID60 In your opinion, in what order do the following groups influence your decision to use **knowledge based on patient experience** in your clinical practice?

______ Patients (1)

______ Patients' family members (2)

______ Naturopathic practitioners (3)

______ Naturopathic professional bodies (e.g. associations) (4)

______ Naturopathic regulatory bodies (5)

______ Government agencies (6)

______ Researchers (7)

______ Third party funders (e.g. health insurers) (8)

______ Other conventional medicine health professionals or organisations (9)

______ Other traditional and complementary medicine health professionals or organisations (10)

| 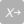 |
| --- |

QID61 In your opinion, in what order to the following groups influence your decision to use **knowledge based on clinical experience** in your clinical practice?

______ Patients (1)

______ Patients' family members (2)

______ Naturopathic practitioners (3)

______ Naturopathic professional bodies (e.g. associations) (4)

______ Naturopathic regulatory bodies (5)

______ Government agencies (6)

______ Researchers (7)

______ Third party funders (e.g. health insurers) (8)

______ Other conventional medicine health professionals or organisations (9)

______ Other traditional and complementary medicine health professionals or organisations (10)

End of Block: Perceived stakeholder influence of knowledge use

Start of Block: Barriers to use of different knowledge types

QID73 The following questions relate to barriers to your use of **research evidence** in your practice

| 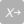 |
| --- |

QID138 In your opinion, to what degree does **lack of time** prevent you from using research evidence in your clinical practice?

- Not a barrier (1)
- A minor barrier (2)
- A moderate barrier (3)
- A major barrier (4)

| 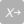 |
| --- |

QID139 In your opinion, to what degree does **lack of resources (ie: access to a computer, the internet or online databases)** prevent you from using research evidence in your clinical practice?

- Not a barrier (1)
- A minor barrier (2)
- A moderate barrier (3)
- A major barrier (4)

| 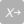 |
| --- |

QID140 In your opinion, to what degree does **lack of clinical research in naturopathy** prevent you from using research evidence in your clinical practice?

- Not a barrier (1)
- A minor barrier (2)
- A moderate barrier (3)
- A major barrier (4)

| 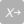 |
| --- |

QID141 In your opinion, to what degree does **insufficient skills for locating research** prevent you from using research evidence in your clinical practice?

- Not a barrier (1)
- A minor barrier (2)
- A moderate barrier (3)
- A major barrier (4)

| 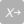 |
| --- |

QID142 In your opinion, to what degree does **insufficient skills for interpreting research** prevent you from using research evidence in your clinical practice?

- Not a barrier (1)
- A minor barrier (2)
- A moderate barrier (3)
- A major barrier (4)

| 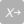 |
| --- |

QID143 In your opinion, to what degree does **insufficient skills to critically appraise/evaluate research** prevent you from using research evidence in your clinical practice?

- Not a barrier (1)
- A minor barrier (2)
- A moderate barrier (3)
- A major barrier (4)

| 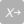 |
| --- |

QID144 In your opinion, to what degree does **insufficient skills to apply research findings to clinical practice** prevent you from using research evidence in your clinical practice?

- Not a barrier (1)
- A minor barrier (2)
- A moderate barrier (3)
- A major barrier (4)

| 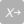 |
| --- |

QID145 In your opinion, to what degree does **lack of incentive to use research evidence** prevent you from using research evidence in your clinical practice?

- Not a barrier (1)
- A minor barrier (2)
- A moderate barrier (3)
- A major barrier (4)

| 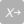 |
| --- |

QID146 In your opinion, to what degree does **lack of personal interest in research evidence** prevent you from using research evidence in your clinical practice?

- Not a barrier (1)
- A minor barrier (2)
- A moderate barrier (3)
- A major barrier (4)

| 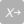 |
| --- |

QID147 In your opinion, to what degree does **lack of relevance to naturopathy** prevent you from using research evidence in your clinical practice?

- Not a barrier (1)
- A minor barrier (2)
- A moderate barrier (3)
- A major barrier (4)

| 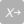 |
| --- |

QID148 In your opinion, to what degree does **lack of colleague support for using research evidence** prevent you from using research evidence in your clinical practice?

- Not a barrier (1)
- A minor barrier (2)
- A moderate barrier (3)
- A major barrier (4)

| 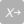 |
| --- |

QID149 In your opinion, to what degree does **lack of industry support for using research evidence** prevent you from using research evidence in your clinical practice?

- Not a barrier (1)
- A minor barrier (2)
- A moderate barrier (3)
- A major barrier (4)

| 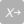 |
| --- |

QID150 In your opinion, to what degree does **patient preference for specific treatments** prevent you from using research evidence in your clinical practice?

- Not a barrier (1)
- A minor barrier (2)
- A moderate barrier (3)
- A major barrier (4)

| Page Break |  |
| --- | --- |

QID151 The following questions relate to potential barriers to your use of **traditional naturopathic knowledge** in your clinical practice.

|  |
| --- |

QID152 In your opinion, to what degree does **lack of time** prevent you from using traditional naturopathic knowledge in your clinical practice?

- Not a barrier (1)
- A minor barrier (2)
- A moderate barrier (3)
- A major barrier (4)

|  |
| --- |

QID153  In your opinion, to what degree does **lack of resources (ie: access to traditional naturopathic texts)** prevent you from using traditional naturopathic knowledge in your clinical practice?

- Not a barrier (1)
- A minor barrier (2)
- A moderate barrier (3)
- A major barrier (4)

|  |
| --- |

QID154 In your opinion, to what degree does **lack of clinical research in naturopathy** prevent you from using traditional naturopathic knowledge in your clinical practice?

- Not a barrier (1)
- A minor barrier (2)
- A moderate barrier (3)
- A major barrier (4)

|  |
| --- |

QID155 In your opinion, to what degree does **insufficient skills for locating**traditional naturopathic knowledge prevent you from using traditional naturopathic knowledge in your clinical practice?

- Not a barrier (1)
- A minor barrier (2)
- A moderate barrier (3)
- A major barrier (4)

|  |
| --- |

QID156 In your opinion, to what degree does **insufficient skills for interpreting traditional naturopathic knowledge** prevent you from using traditional naturopathic knowledge in your clinical practice?

- Not a barrier (1)
- A minor barrier (2)
- A moderate barrier (3)
- A major barrier (4)

|  |
| --- |

QID157 In your opinion, to what degree does **insufficient skills to critically appraise/evaluate traditional naturopathic knowledge** prevent you from using traditional naturopathic knowledge in your clinical practice?

- Not a barrier (1)
- A minor barrier (2)
- A moderate barrier (3)
- A major barrier (4)

|  |
| --- |

QID158 In your opinion, to what degree does **insufficient skills to apply traditional naturopathic knowledge to clinical practice** prevent you from using traditional naturopathic knowledge in your clinical practice?

- Not a barrier (1)
- A minor barrier (2)
- A moderate barrier (3)
- A major barrier (4)

|  |
| --- |

QID159 In your opinion, to what degree does **lack of incentive to use traditional naturopathic knowledge** prevent you from using traditional naturopathic knowledge in your clinical practice?

- Not a barrier (1)
- A minor barrier (2)
- A moderate barrier (3)
- A major barrier (4)

|  |
| --- |

QID160 In your opinion, to what degree does **lack of personal interest in traditional naturopathic knowledge** prevent you from using traditional naturopathic knowledge in your clinical practice?

- Not a barrier (1)
- A minor barrier (2)
- A moderate barrier (3)
- A major barrier (4)

|  |
| --- |

QID161 In your opinion, to what degree does **lack of relevance to modern-day naturopathic practice** prevent you from using traditional naturopathic knowledge in your clinical practice?

- Not a barrier (1)
- A minor barrier (2)
- A moderate barrier (3)
- A major barrier (4)

|  |
| --- |

QID162 In your opinion, to what degree does **lack of colleague support for using traditional naturopathic knowledge** prevent you from using traditional naturopathic knowledge in your clinical practice?

- Not a barrier (1)
- A minor barrier (2)
- A moderate barrier (3)
- A major barrier (4)

|  |
| --- |

QID163 In your opinion, to what degree does **lack of industry support for using traditional naturopathic knowledge** prevent you from using traditional naturopathic knowledge in your clinical practice?

- Not a barrier (1)
- A minor barrier (2)
- A moderate barrier (3)
- A major barrier (4)

|  |
| --- |

QID164 In your opinion, to what degree does **patient preference for specific treatments** prevent you from using traditional naturopathic knowledge in your clinical practice?

- Not a barrier (1)
- A minor barrier (2)
- A moderate barrier (3)
- A major barrier (4)

| Page Break |  |
| --- | --- |

QID166 The following questions relate to potential barriers to your use of **knowledge from patient experiences or preferences** in your clinical practice.

|  |
| --- |

QID167 In your opinion, to what degree does **lack of time** prevent you from using knowledge from patient experiences or preferences in your clinical practice?

- Not a barrier (1)
- A minor barrier (2)
- A moderate barrier (3)
- A major barrier (4)

|  |
| --- |

QID168 In your opinion, to what degree does **uncertainty about an individual patient's experiences or preferences** prevent you from using knowledge from patient experiences or preferences in your clinical practice?

- Not a barrier (1)
- A minor barrier (2)
- A moderate barrier (3)
- A major barrier (4)

|  |
| --- |

QID169 In your opinion, to what degree does **insufficient skills for collecting information from patients about their experiences or preferences** prevent you from using knowledge from patient experiences or preferences in your clinical practice?

- Not a barrier (1)
- A minor barrier (2)
- A moderate barrier (3)
- A major barrier (4)

|  |
| --- |

QID170 In your opinion, to what degree does **insufficient skills to critically appraise/evaluate patient experiences or preferences** prevent you from using knowledge from patient experiences or preferences in your clinical practice?

- Not a barrier (1)
- A minor barrier (2)
- A moderate barrier (3)
- A major barrier (4)

|  |
| --- |

QID171 In your opinion, to what degree does **insufficient skills to apply patient experiences or preferences** prevent you from using knowledge from patient experiences or preferences in your clinical practice?

- Not a barrier (1)
- A minor barrier (2)
- A moderate barrier (3)
- A major barrier (4)

|  |
| --- |

QID172 In your opinion, to what degree does **lack of incentive to consider patient experiences or preferences** prevent you from using knowledge from patient experiences or preferences in your clinical practice?

- Not a barrier (1)
- A minor barrier (2)
- A moderate barrier (3)
- A major barrier (4)

|  |
| --- |

QID173 In your opinion, to what degree does **lack of personal interest in patient experiences or preferences** prevent you from using knowledge from patient experiences or preferences in your clinical practice?

- Not a barrier (1)
- A minor barrier (2)
- A moderate barrier (3)
- A major barrier (4)

|  |
| --- |

QID174 In your opinion, to what degree does **lack of relevance to naturopathic practice** prevent you from using knowledge from patient experiences or preferences in your clinical practice?

- Not a barrier (1)
- A minor barrier (2)
- A moderate barrier (3)
- A major barrier (4)

|  |
| --- |

QID175 In your opinion, to what degree does **lack of colleague support for considering patient experiences or preferences** prevent you from using knowledge from patient experiences or preferences in your clinical practice?

- Not a barrier (1)
- A minor barrier (2)
- A moderate barrier (3)
- A major barrier (4)

|  |
| --- |

QID176 In your opinion, to what degree does **lack of industry support for considering patient experiences or preferences** prevent you from using knowledge from patient experiences or preferences in your clinical practice?

- Not a barrier (1)
- A minor barrier (2)
- A moderate barrier (3)
- A major barrier (4)

|  |
| --- |

QID177 In your opinion, to what degree does **differences between patient experiences or preferences and other types of knowledge** prevent you from using knowledge from patient experiences or preferences in your clinical practice?

- Not a barrier (1)
- A minor barrier (2)
- A moderate barrier (3)
- A major barrier (4)

| Page Break |  |
| --- | --- |

QID178 The following questions relate to potential barriers to your use of knowledge from your own clinical experience in your clinical practice.

|  |
| --- |

QID179 In your opinion, to what degree does **lack of time** prevent you from using knowledge from your own clinical experience in your clinical practice?

- Not a barrier (1)
- A minor barrier (2)
- A moderate barrier (3)
- A major barrier (4)

|  |
| --- |

QID180 In your opinion, to what degree does **uncertainty about your clinical experience** prevent you from using knowledge from your own clinical experience in your clinical practice?

- Not a barrier (1)
- A minor barrier (2)
- A moderate barrier (3)
- A major barrier (4)

|  |
| --- |

QID181 In your opinion, to what degree does **insufficient clinical experience** prevent you from using knowledge from your own clinical experience in your clinical practice?

- Not a barrier (1)
- A minor barrier (2)
- A moderate barrier (3)
- A major barrier (4)

|  |
| --- |

QID182 In your opinion, to what degree does **insufficient skills to reflect on your own clinical experience** prevent you from using knowledge from your own clinical experience in your clinical practice?

- Not a barrier (1)
- A minor barrier (2)
- A moderate barrier (3)
- A major barrier (4)

|  |
| --- |

QID183 In your opinion, to what degree does **insufficient skills to apply your clinical experience to clinical decisions** prevent you from using knowledge from your own clinical experience in your clinical practice?

- Not a barrier (1)
- A minor barrier (2)
- A moderate barrier (3)
- A major barrier (4)

|  |
| --- |

QID184 In your opinion, to what degree does **lack of incentive to use your clinical experience** prevent you from using knowledge from your own clinical experience in your clinical practice?

- Not a barrier (1)
- A minor barrier (2)
- A moderate barrier (3)
- A major barrier (4)

|  |
| --- |

QID185 In your opinion, to what degree does **lack of personal interest in using your clinical experience** prevent you from using knowledge from your own clinical experience in your clinical practice?

- Not a barrier (1)
- A minor barrier (2)
- A moderate barrier (3)
- A major barrier (4)

|  |
| --- |

QID186 In your opinion, to what degree does **lack of relevance to naturopathic practice** prevent you from using knowledge from your own clinical experience in your clinical practice?

- Not a barrier (1)
- A minor barrier (2)
- A moderate barrier (3)
- A major barrier (4)

|  |
| --- |

QID187 In your opinion, to what degree does **lack of colleague support for using your clinical experience** prevent you from using knowledge from your own clinical experience in your clinical practice?

- Not a barrier (1)
- A minor barrier (2)
- A moderate barrier (3)
- A major barrier (4)

|  |
| --- |

QID188 In your opinion, to what degree does **lack of industry support for using your clinical experience** prevent you from using knowledge from your own clinical experience in your clinical practice?

- Not a barrier (1)
- A minor barrier (2)
- A moderate barrier (3)
- A major barrier (4)

|  |
| --- |

QID189 In your opinion, to what degree does **differences between your clinical experience and other types of knowledge** prevent you from using knowledge from your own clinical experience in your clinical practice?

- Not a barrier (1)
- A minor barrier (2)
- A moderate barrier (3)
- A major barrier (4)

End of Block: Barriers to use of different knowledge types

**Thank you note for Excluded Participants**

Thank you for your interest in participating in this study. Unfortunately, you do not meet our inclusion criteria which requires participants be in clinical practice within the last 12 months. 

Thank you again.

Regards
Dr Amie Steel (on behalf of the research team)

**Thank you note for Completed Participants**

Thank you so much for finishing the survey! Your responses will be combined with answers from naturopaths around the world and published and shared widely. 

You are eligible to enter the draw to win one of five naturopathic clinical text books. Just click this [link](https://utsau.au1.qualtrics.com/jfe/form/SV_bKGDePTQyplkIOp) which will take you to a new page where you can enter your contact information. None of your responses to this survey will be linked to your contact information so your answers will remain anonymous.

**Thank you note for Prize Draw entry**

Thank you again for participating in our study. 

If you have any questions about this study or the prize draw please contact the study team on amie.steel@uts.edu.au or 0418 786 186
